# Understanding the impact of dog ownership on autistic adults: implications for mental health and suicide prevention

**DOI:** 10.1038/s41598-021-02504-8

**Published:** 2021-12-08

**Authors:** Ana Maria Barcelos, Niko Kargas, Chris Packham, Daniel S. Mills

**Affiliations:** 1 School of Life Sciences, University of Lincoln, Lincoln, UK; 2 School of Psychology, University of Lincoln, Lincoln, UK

**Keywords:** Psychology, Anxiety, Autism spectrum disorders, Depression, Animal behaviour

## Abstract

Mental health problems and suicide are more frequent in autistic adults than general population. Dog ownership can improve human well-being. This study aimed to generate a framework of well-being outcomes for dog-related activities in autistic adults and compare it to the framework generated for a general adult population. Thirty-six autistic dog owners (18–74 years old, 18 males) from diverse UK regions were interviewed and transcripts thematically analysed. 16.7% reported that their dogs prevented them from taking their own lives, mainly due to the dog's affection and the need to care for the animal. Close dog-owner interactions (e.g., cuddling, walking, dog's presence) were the most frequent activities improving emotions/moods and life functioning, whereas routine-like activities (e.g., feeding the animal) particularly enhanced life functioning. Well-being worsening was mainly linked to dog behaviour problems, dog poor health/death and obligations to the dog. Despite some negatives associated with ownership, having a dog could improve the well-being of many autistic adults and assist suicide prevention strategies in this high-risk group. The framework was consistent with that generated previously, indicating its robustness and the potential opportunity to focus on dog-related activities rather than the vague concept of “ownership” when considering the impact of ownership on well-being.

## Introduction

Although the recognised prevalence of autistic individuals is increasing^1^, with a figure of > 1% for the adult population in England^2^, there is still limited understanding of the condition and acceptance by the public^3,4^. There is a disproportionate amount of research focusing on children^5^, and a common misunderstanding is that autism, a lifelong neurodevelopmental condition^6^, is only present during childhood^5,7^. Autism in adulthood is accompanied by mental health problems, in a high proportion of cases^8^. While 25% of the general population of adults in the UK suffers a diagnosable mental health problem^9^, these problems affect nearly 80% of those on the autism spectrum; depression and anxiety being the leading mental health issues experienced by these individuals^10^. Furthermore, adults on the spectrum are at much higher risk of attempting suicide^11,12^ and having suicidal thoughts (e.g., 66% versus 17% suicidal ideation)^13,14^ than the general population (for a review see^15^). Suicide is a leading cause of premature death in autistic people^16^. Therefore, it is crucial to better understand the factors that improve or worsen the well-being of autistic adults and develop prevention strategies for suicide in this population^14,17^.

Owning a dog has been repeatedly suggested to protect and/or improve people's well-being^18–21^, even under more challenging circumstances, such as the COVID-19 pandemic^22–24^, a positive diagnosis of HIV^25–27^, old age^28–30^, chronic pain^31–34^, etc. In general terms, owning a dog seems to have the potential to improve the quality of life of any person (who likes dogs). In relation to autism, dogs can be helpful in a variety of ways. Autism assistance dogs are trained to engage in specific assistance-related activities which have been identified as being very helpful for their owners^35^. However, their value clearly extends beyond this and the companionship provided by these dogs and pet dogs may also bring a range of positive benefits to individuals with autism and their wider family. In prospective case control studies with families with autistic children, acquiring a pet dog has been shown to decrease the child's anxiety^36^, family difficulties^36,37^ and parenting stress^37,38^. Qualitative studies with parents of autistic children have suggested that pet dog ownership improves family functioning^39^, child social and emotional skills^39,40^, confidence, practice of physical activities^39^ and provides companionship, safety and calmness/stress relief to the child^40^. Likewise, in a recent survey with hundreds of parents of autistic children, more than 50% of them reported that their pet (mostly dogs and cats) helps increase their child's relaxation and companionship and affection for their child^41^. However, despite the suggested benefits of dog ownership for the general adult population and for autistic children, little is known about the potential mechanism behind this effect (or even if it is dog-specific) and the impact of dog ownership on adults on the autism spectrum.

Barcelos et al*.*^42^ recently proposed a comprehensive framework for dog–human related activities and their impact on hedonic well-being, eudaimonic well-being and life satisfaction among the general adult population of dog owners in the UK. However, whether this framework applies to autistic individuals remains unknown. 'Dog–human related activities' (referred to simply as 'activities' from here-on) are the various activities/events that occur due to the existence of a dog in someone's life, and it includes events that happen whether the dog is present (e.g., petting the dog; going to the vet; the dog barking) or not (e.g., doing a dog training course online, reading a book on dog behaviour, posting pictures of the dog on social media^42^). For the purposes of our work, hedonic well-being consists of positive and negative affect (emotions, moods); while life satisfaction is the perception of one's own life, e.g. feeling good or bad about it^43^; and eudaimonic well-being describes the functioning of a person in relation to six areas: (1) autonomy—independence and freedom from others’ approval -, (2) environmental mastery—management of the surrounding environment to fit one's needs -, (3) personal growth,—self-realization and achievement of personal potential, (4) positive relations with others—good social relations, empathic and affectionate feelings for others and, (5) purpose in life—meaning in life and understanding of own purpose -, (6) self-acceptance—positive self-evaluation with acceptance of past life and good and bad qualities^44^.

In the current study, semi-structured interviews were conducted with autistic adults who own a dog and live in the UK, in order to evaluate to what extent the framework developed by Barcelos et al*.*^42^ can be applied to autistic adults.

## Methods

### Ethical approval

The study was approved by the ethical committee of the University of Lincoln (reference 2020_0503) and was in accordance with both the university Research Ethics Policy and the BPS Code of Ethics and Conduct. Prior to the interviews, all participants were given detailed information about the study, data collection/usage and structure of the interview, and their electronic informed consent was obtained.

### Participants

To be considered for participation in the study candidates needed to own a dog, be at least 18 years old, have a formal diagnosis of autism spectrum disorder (i.e., diagnosed by a psychologist, psychiatrist, or another health carer) categorised as "high-functioning autism" or "Asperger syndrome", live in the UK and have access to the internet. Recruitment occurred through national/regional autism organisations, social media (Facebook groups of autism and personal contacts) and media reports. Interested candidates filled an online recruitment form on Qualtrics^XM^. Information about themselves (e.g., demographics, mental health) and their dogs (e.g., age, size) was collected to help in a purposeful sampling process^45^, to gain a balanced diverse sample in terms of owner and dog characteristics. Through this process, the authors selected participants (Supplementary Table S2) from an initial sample of 198 eligible candidates (median age 25–34 years old, ranging from 18–24 to 65–74). Sampling stopped after the 36th interview, due to inductive thematic saturation, which is the non-emergence of new themes/codes^46^. Participants (Supplementary Table S2) were equally balanced in sex (18 each), aged from 18–24 to 65–74 years old (median 25–34), lived in various regions of the UK, varied in levels of depression and anxiety, and owned dogs of diverse characteristics (e.g., age, sex, size). Depression and anxiety were screened using the Patient Health Questionnaire (PHQ-9^47^) and the Generalized Anxiety Disorder scale (GAD-7^48^), respectively. Most of the participants (22, 61.1%) were moderately to severely depressed or anxious, and 13 (36.1%) had moderate to severe levels of both depression and anxiety. Each of the 36 selected participants received a £14 Gift Card as a token recognition of their contribution to the study.

### Interviews

After a pilot study with one adult dog owner on the autism spectrum, semi-structured interviews^49^ with each of the 36 participants were conducted by an experienced interviewer (AMB) following a similar methodology to Barcelos et al*.*^42^. Due to the COVID-19 pandemic remote one-to-one interviews on Blackboard Collaborate Ultra, a real-time video conferencing tool were used in place of live meetings. On average, interviews lasted 29 min and ranged from 15 to 49 min. AMB guided the conversation with the aid of a PowerPoint presentation, shared with the participant, and a pre-designed interview guide (available in the Supplementary Information file, adapted from Barcelos et al*.*^42^) composed of five main steps. First, the interviewer briefly explained the procedures of the interview and started recording the meeting. Second, the interviewer introduced the concept of dog–human related activity (both direct and indirect activities^42^). Third, the basic concepts of hedonic well-being and life satisfaction were explained, with simple terminology (e.g., instead of saying 'affect'/'hedonic', the words 'feeling', 'emotion', 'mood' were used), and the participant was asked to say the four most important dog–human related activities in his/her life that had an impact on his/her hedonic well-being or life satisfaction. Fourth, the interviewer explained the concept of eudaimonic well-being using simplified terminology and asked the participants to say the four most important dog–human related activities in his/her life that had an impact on any of the six elements of eudaimonic well-being described above. Finally, the interviewer closed the meeting acknowledging the participant for his/her participation. In steps three and four, where participants talked about dog–human related activities and the impact they have on their well-being, prompts and probes were used by the interviewer to gather further information, clarify statements^50,51^, and facilitate the collection of additional data (more than eight dog–human related activities per participant).

### Transcription and data analysis

Transcriptions were conducted on Otter transcription software. The researcher listened to the audio recordings and wrote/edited the transcripts simultaneously. Data analysis was performed on NVivo 11 and followed the same methodology of Barcelos et al*.*^42^. First, dog–human related activities and their reported well-being outcomes were coded. Second, similar activity codes were grouped together, such as both 'dog barking' and 'dog pulling on the lead' within the theme 'Unwanted behaviours/situations'. Hedonic outcomes (e.g., happy, calm, angry, sad) were placed into one of four themes (positive valence-high arousal; positive valence-low arousal; negative valence-high arousal; negative valence-low arousal) based on the dimensional models of affect of Russel^52^, Scherer^53^, and Yik et al*.*^54^. Valence indicates the pleasantness (positiveness) or unpleasantness (negativity) of the affect and arousal represents its energy/mobilisation. Eudaimonic outcomes (e.g., 'more purpose in life, 'improved relationship with other people') were grouped together according to Ryff's^55^ six-element classification. Third, matrix coding (cross-tabulation) was conducted to see how frequently each dog–human related activity was linked to a specific well-being outcome (e.g., 'dog greeting the owner' was reported N times to increase the hedonic theme 'positive arousal-high valence'). Finally, a heat map was created on Excel to facilitate the interpretation of the matrix coding.

## Results

**Figure 1:**
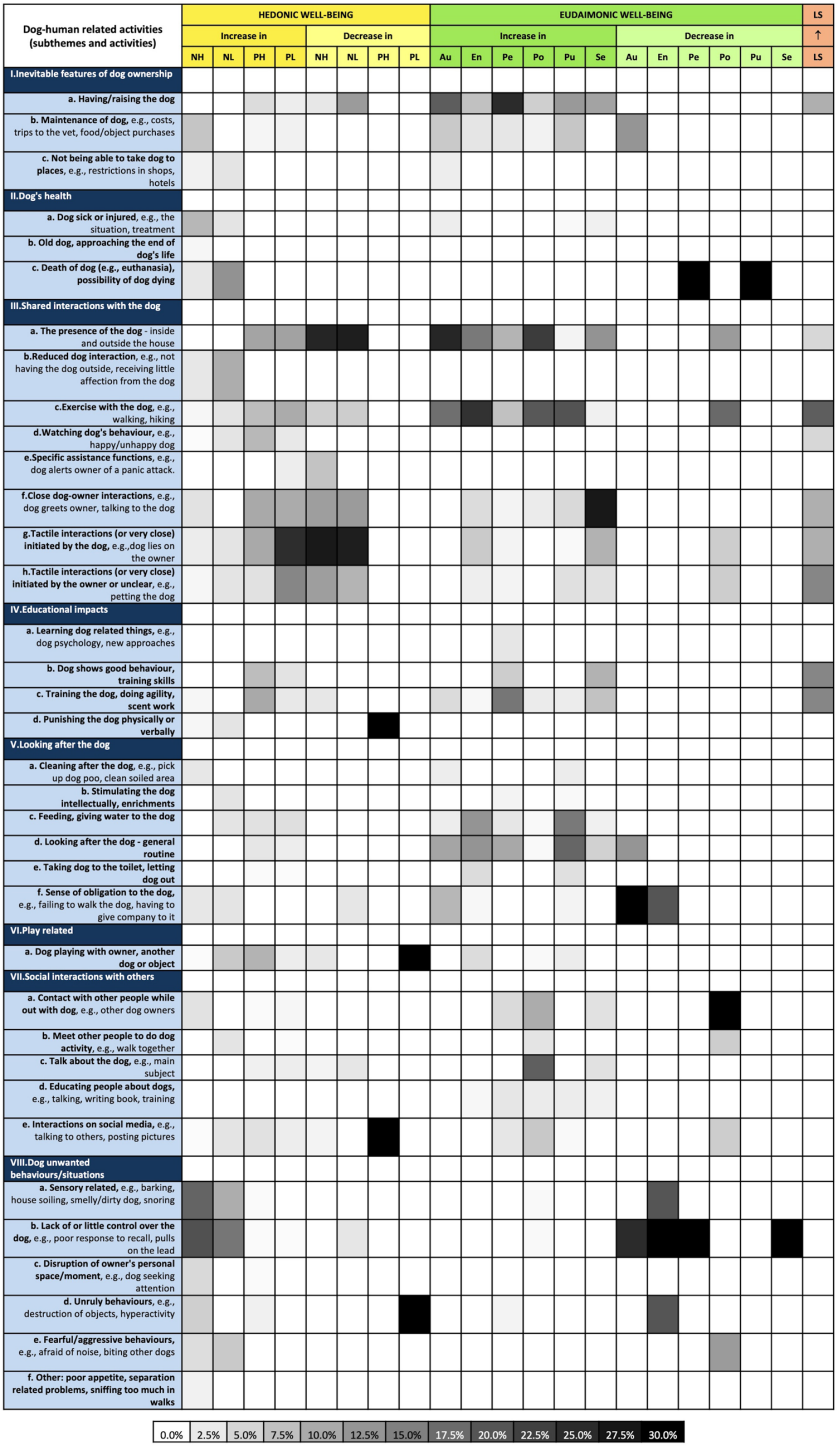
Heat map of dog–human related activities and their respective well-being outcomes. Darker cells represent a higher proportion of mentions of an activity (row) in a respective well-being outcome (column). E.g., ‘the presence of the dog' (dark cell) was mentioned to increase autonomy more times than ‘training the dog’ (light cell). N and P (negative and positive valence, respectively), H and L (high and low arousal, respectively), Au (autonomy), En (environmental mastery), Pe (personal growth), Pu (purpose in life), Po (positive relations), Se (self-acceptance), LS (life satisfaction increase).

Based on interviews with a heterogeneous sample of 36 autistic adults who own a dog and live in the UK, a total of 1243 mentions of activities and their respective well-being outcomes were coded, leading to the creation of a framework of 85 dog–human related activities and their reported well-being outcomes (Supplementary Fig. S1). Through thematic analysis, the activities were grouped into eight themes and 36 subthemes, which can be seen in both the simplified version of the framework (Fig. 1) and its full version (Supplementary Fig. S1). The framework provides an overview of the impact of activities on aspects of hedonic well-being, eudaimonic well-being and life satisfaction. For example, when looking at “increase in purpose in life” (column 'Pu', Fig. 1), the subtheme 'exercise with the dog' is darker than 'dog playing with owner[…]', indicating that exercising the dog was more frequently reported to improve owners' purpose in life than dog playing. Further details of the framework are provided below.

### Overview of the activities and well-being outcomes of the framework

'Shared interactions with the dog' (567 mentions, 45.6%) was the theme of activity most frequently described to impact participants' well-being, being followed by 'looking after the dog' (156, 12.6%), 'dog unwanted behaviours/situations' (133, 10.7%), 'inevitable features of dog ownership' (123, 9.9%), 'social interactions with others' (109, 8.8%), 'educational impacts' (90, 7.2%), 'play related' (33, 2.7%) and 'dog's health' (32, 2.6%). Among the themes, 'unwanted behaviours/situations' and 'dog's health' had notably negative impacts on participant well-being, with more than 90% of their mentions being linked to negative well-being outcomes; whereas the other themes were generally positive (6.0–16.5% of mentions linked to negative outcomes).

Overall, positive well-being outcomes were much more frequently linked to dog–human related activities than negative well-being outcomes, the first accounting for 80.0% of all the mentions. When comparing areas of well-being, emotions and moods (hedonic well-being) were the aspects of well-being most frequently mentioned by the participants (664 mentions, 53.4%) to be impacted by dog–human related activities. This was followed by eudaimonic well-being, which was also highly prevalent in the interviews (558, 44.9%). Life satisfaction was the least reported, being poorly linked to dog–human related activities, with just 21 mentions (1.7%).

### Themes of activities reported to impact on emotions/moods (hedonic well-being), life functioning (eudaimonic well-being) and life satisfaction

**Figure 2:**
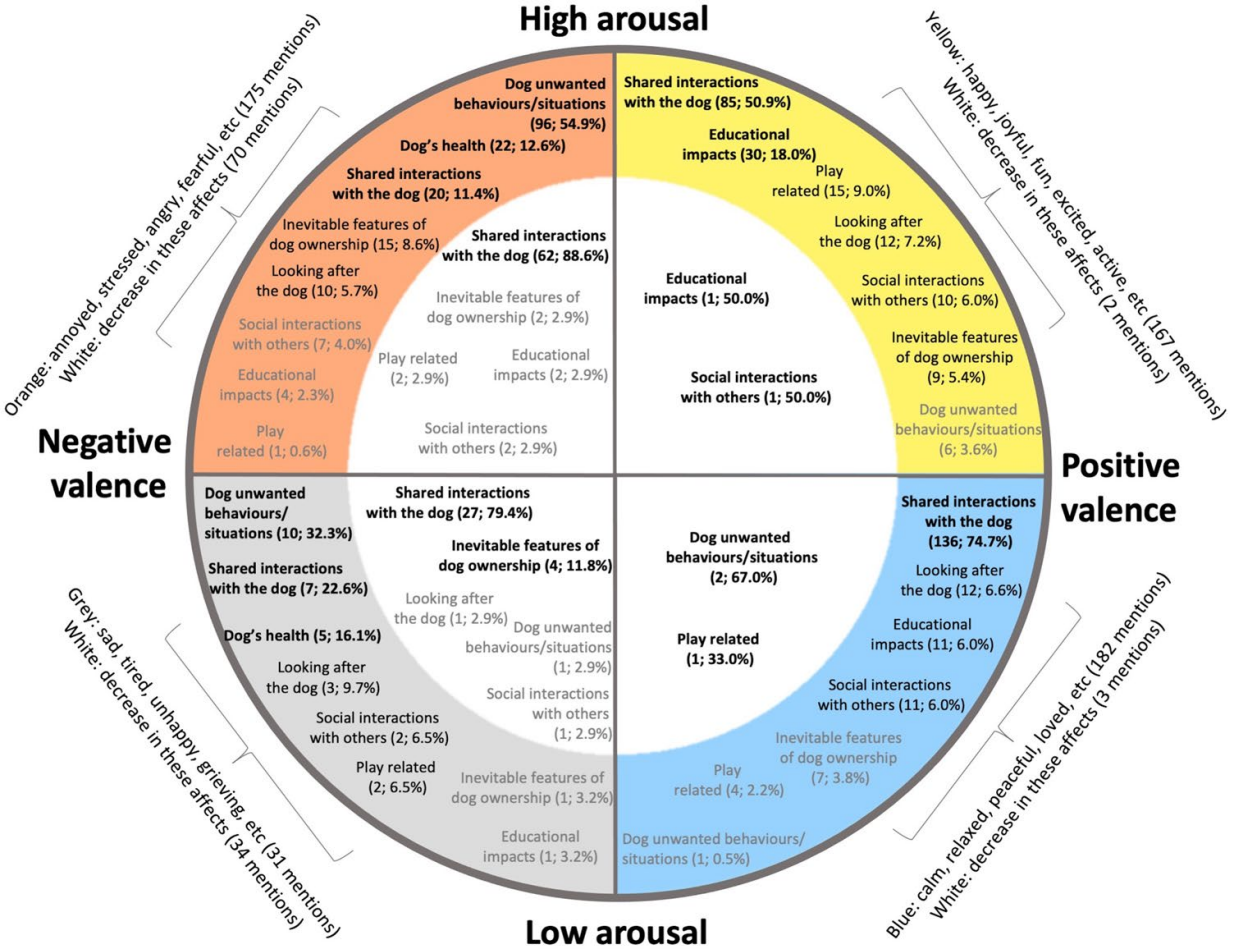
Reported hedonic well-being outcomes of themes of activities across a multidimensional model of affect. Themes in coloured areas increase the quadrant's mood/emotions, whereas themes in white areas decrease that quadrant's affect. The spatial position of themes within the same quadrant do not indicate its intensity. The numbers next to the themes indicate the number of times (and percentage) a theme was reported to generate the respective well-being outcome. Themes in bold**:** were frequently mentioned to impact on the area where it is located (10% or more). Black: moderate frequency of mentions (5.0–9.9%). Grey: low frequency of mentions (0.01–4.9%).

**Figure 3:**
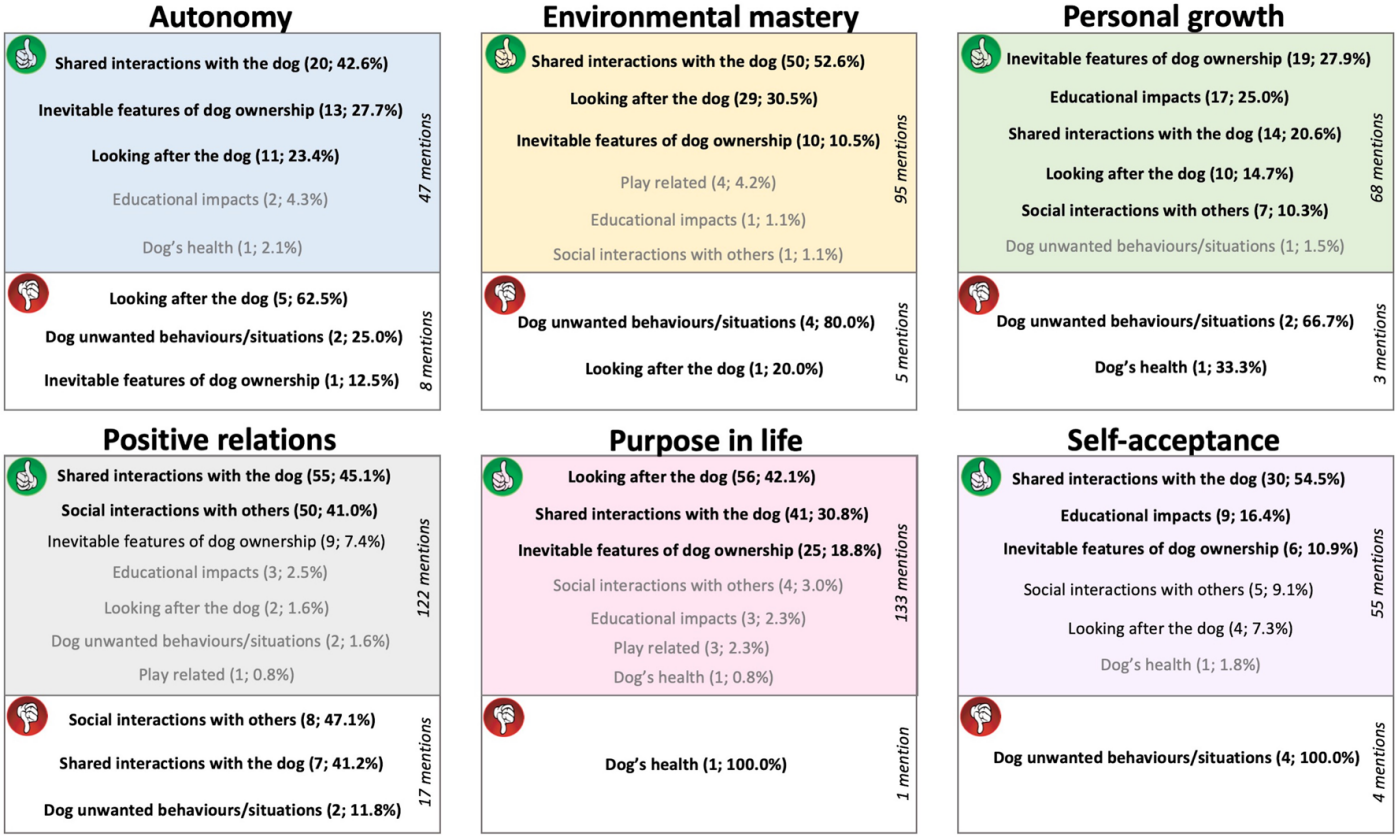
Reported eudaimonic well-being outcomes of themes of activities across six areas of life functioning. The numbers next to the themes indicate the number of times (and percentage) a theme was reported to generate the respective well-being outcome. Themes in bold: frequently mentioned in the respective element of well-being (10% or more). Black: moderate frequency of mentions (5.0–9.9%). Grey: low frequency of the mentions (0.01–4.9%). Activities placed in the green ‘thumbs-up’ area are beneficial to the referred well-being, while those in red ‘thumbs-down’ areas inhibit that aspect of well-being.

An overview of all themes and their respective impact on each aspect of hedonic and eudaimonic well-being is provided in Figs. 2 and 3, respectively. Figure 2 makes analogy with dimensional models of affect (Russel^52^, Scherer^53^, and Yik et al*.*^54^). Shared interactions with the dog, such as physical contact, the mere presence of the dog and exercising together, were repeatedly described to improve participants' moods and emotions (Fig. 2). Its relevance is noticed in all positive elements of hedonic well-being (e.g., increase in positive valence-high arousal, decrease in negative valence-high arousal), making participants happier, calmer, but also less stressed and sad. With regards to the worsening of moods and emotions, unwanted behaviours/situations caused by the dog were the most relevant, not only increasing negative affect (e.g., more stress/anger and sadness), but also decreasing participants' positive affect (e.g., less relaxation).

With regards to eudaimonic well-being, shared interactions with the dog, inevitable features of dog ownership (e.g., having the dog) and looking after the animal were predominantly described to improve its various aspects, such as purpose in life, autonomy and environmental mastery (Fig. 3). In contrast, dog's unwanted behaviours/situations prevailed among the themes of activities responsible for worsening participants' eudaimonic well-being. Further information is provided in the six squares of Fig. 3, which represent the six elements of eudaimonic well-being^55^.

Finally, life satisfaction was only described in relation to three themes of activities: shared interactions with the dog (13 mentions, 61.9%, e.g., dog walks), educational impacts (6 mentions, 28.6%, e.g., dog training), and inevitable features of dog ownership (2 mentions, 9.5%, e.g., having the dog). Worsening of life satisfaction due to the dog was not reported by any participant.

### Activities reported to impact on emotions/moods (hedonic well-being)

The activities most frequently reported to improve and worsen specific emotions/moods in autistic adults are summarised in Tables 1 and 2, respectively. The remaining activities are described in Supplementary Figure S1.

**Table 1:** Activities reported to improve emotions/moods of autistic adults.

	Most frequent subthemes (≥ 5% mentions)	Sample extract from interviews
**Increase in**		
**Positive valence-high arousal,** e.g., happy, excited, joyful, fun 167 mentions	The presence of the dog (18; 10.8%) Training the dog, doing agility, scent work (17; 10.2%) Close dog-owner interactions (17; 10.2%) Tactile interactions initiated by the dog (17; 10.2%) Dog playing (15; 9.0%) Watching dog's behaviour (14; 8.4%) Exercise with the dog (13; 7.8%) Dog shows good behaviour, training skills (13; 7.8%)	*"She likes playing with balls and that kind of thing, that's quite a fun activity to deal with it, gets me out of the house, which I wouldn't normally do that much. It's quite happy, that kind of thing." P3*
**Positive valence-low arousal,** e.g., calm, relaxed, peaceful 182 mentions	Tactile interactions initiated by the dog (45; 24.7%) Tactile interactions initiated by the owner or unclear (26; 14.3%) The presence of the dog (20; 11.0%) Exercise with the dog (18; 9.9%) Close dog-owner interactions (18; 9.9%)	*"She comes over like that, and then lies on me. I find that the combination of stroking her and her lying on me it's almost like having your personal kind of heated weighted blanket. It's just really calming. I feel so relaxed and just content when she's like lying on me and I'm stroking that soft bit of fur on her chest." P1*
**Decrease in**		
**Negative valence-high arousal,** e.g., stressed, angry, frustrated, worried, annoyed 70 mentions	Tactile interactions initiated by the dog (19; 27.1%) The presence of the dog (18; 25.7%) Close dog-owner interactions (8; 11.4%) Tactile interactions initiated by the owner or unclear (8; 11.4%) Specific assistance functions (5; 7.1%) Exercise with the dog (4; 5.7%)	*"If I have a meltdown, or if I'm really stressed, she sometimes senses it. And if I'm gonna hurt myself or anything, she will just sort of like jump on my lap or jump on the bed and give me that look—come on, have a cuddle. And sort of just lay there for a second, you know, to call me and stuff. And it can help because she's not talking to me. She's just there" P25*
**Negative valence-low arousal,** e.g., sad, tired, low, depressed 34 mentions	The presence of the dog (9; 26.5%) Tactile interactions initiated by the dog (9; 26.5%) Having/raising the dog (4; 11.8%) Close dog-owner interactions (4; 11.8%) Tactile interactions initiated by the owner or unclear (3; 8.8%) Exercise with the dog (2; 5.9%)	*"Whenever I'm feeling sad or depressed or panicking myself, just having one of the dogs nearby is almost a calming presence. It's a feeling of—it's going to be okay. It's a really weird thing to say, but it's a bond, I suppose you could say that, that's a very positive thing, I think." P26*

The numbers next to the subthemes indicate the number of times (and percentage) a subtheme was reported to generate the respective well-being outcome.

**Table 2:** Activities reported to worsen emotions/moods of autistic adults.

	Most frequent subthemes (≥ 5% mentions)	Sample extract from interviews
**Increase in**		
**Negative valence-high arousal,** e.g., stressed, angry, frustrated, worried, annoyed 175 mentions	Lack of or little control over the dog (36; 20.6%) Sensory related (32; 18.3%) Dog sick or injured (15; 8.6%) Maintenance of dog (12; 6.9%) Unruly behaviours (10; 5.7%)	*"If she's barking at the cat in the garden or something, that doesn't bother me, but if she's right next to me and there's no warning, and she just barks, I burst sometimes into tears or I just jump by and my whole body goes into like some kind of spasm. It's just intense stress" P1*
**Negative valence-low arousal,** e.g., sad, tired, low, depressed 31 mentions	Lack of or little control over the dog (5; 16.1%) Death of dog, possibility of dog dying (4; 12.9%) Reduced dog interaction (3; 9.7%) Sensory related (3; 9.7%) Fearful/aggressive behaviours (2; 6.5%) Dog playing (2; 6.5%)	*"I'm very conscious of the fact that they [dogs] are not always going to be around. And it upsets me looking at them knowing that I've only got a limited amount of time with them. It's really sad, distraught to be honest. I want to cry if I think about it." P21*
**Decrease in**		
**Positive valence-high arousal,** e.g., happy, excited, joyful, fun 2 mentions	Punishing the dog physically or verbally (1; 50.0%) Interactions on social media (1; 50.0%)	*"My Bulldog is quite skinny, he's quite fit and lots of other dogs can be quite overweight. And I posted a photo of him on social media and I got absolutely slated […] that does necessarily knock my confidence, obviously" P27*
**Positive valence-low arousal,** e.g., calm, relaxed, peaceful 3 mentions	Unruly behaviours (2; 66.7%) Dog playing (1; 33.3%)	*"Sometimes you just want a little bit of peaceful quiet to read a book or watch your favourite TV programme. Then he likes to come along and nibble away at your feet and steal your shoes and runs away with them. He is a puppy, so when you want to have a piece of quiet, he's not thinking the same as you." P36*

The numbers next to the subthemes indicate the number of times (and percentage) a subtheme was reported to generate the respective well-being outcome.

The mere presence of the dog, tactile interactions, walking the dog and other close interactions with the animal (e.g., being greeted by dog) improved all aspects of participants' hedonic well-being (Table 1). Besides these, playing with the dog and training the dog were particularly prominent among improvements in positive emotions/moods of high arousal; specific assistance functions (e.g., dog alerting owner of a panic attack) were important for the reduction of negative affect of high arousal; and simply having the dog help owners feel less sad, depressed or low (negative valence low arousal).

Failing to control the dog (e.g., dog not responding to owner's recall while out in a park, pulling a lot on the lead) and being exposed to sensory-related unwanted behaviours/situations (e.g., dog barking, house soiling, dog rolls in fox faeces) were related to a deterioration of emotions and moods in autistic dog owners (Table 2). Participants also said they become worried and stressed when their dogs were sick or injured, or when they had to deal with the maintenance of the animal, especially seeing the vet and managing the costs of the dog. In contrast, low negative emotions/moods, such as sadness, were reported to increase when their dog died or was at risk of dying, and also due to reduced interaction with the animal (e.g., the owner is sad/low for not having the company of his dog while outside).

### Activities reported to affect life functioning (eudaimonic well-being)

Four activities were repeatedly mentioned to improve most aspects of participants' eudaimonic well-being: (1) the routine of looking after the animal, (2) being with the dog / the dog's presence, (3) walking the dog, and (4) simply having the dog (Table 3). Participants felt more autonomous and in control of their environment in the company of their dogs, particularly outside (e.g., being able to shop, walk alone, travel), and also from being a dog owner and caring for the animal on a regular basis. Personal growth was particularly linked to a sense of achievement, not only for being a good dog carer, but also for training the animal and witnessing the dog's good behaviour. Unsurprisingly, positive relations with others improved with dogs seen to act as a social lubricant for the instigation of contact (e.g., people approaching dog owners because of the dog). Notably, dogs were also reported to boost the confidence of their owners in those social interactions, forming the main topic of conversation. Purpose in life was directly linked to the routine of looking after the animal (e.g., food, water, walks) and the responsibility of being an owner. Improvements in owners' self-acceptance, finally, were related to the dogs' manifestations of affection towards their owners, such as greeting and touching the owner, and also to a sense of accomplishment for having a well-behaved dog and being able to effectively train and care for the animal.

**Table 3:** Activities described to improve aspects of eudaimonic well-being.

	Most frequent subthemes (≥ 5% mentions)	Sample extract from interviews
**Increase in**		
**Autonomy ** 49 mentions	The presence of the dog (12; 25.5%) Having/raising the dog (9; 19.1%) Exercise with the dog (8; 17.0%) Looking after the dog—general routine (5; 10.6%) Sense of obligation to the dog (4; 8.5%) Maintenance of dog (3; 6.4%)	*"I think my dog helps me, because now I can go out on my own, I can do like the food shopping and things like that on my own. Whereas before I found that really hard. Her company makes me feel a bit more confident, I think and less worried about things" P5*
**Environmental mastery** 95 mentions	Exercise with the dog (23; 24.2%) The presence of the dog (15; 15.8%) Feeding, giving water to the dog (12; 12.6%) Looking after the dog—general routine (12; 12.6%) Having/raising the dog (7; 7.4%) Tactile interactions initiated by the dog (6; 6.3%)	*"I was in a pretty bad state before. Like I didn't really clean up the house[…]. Then I got him, and I need to clean up the house[…], I need to get up in the morning to feed him and take him out for a walk. And it suddenly gave me all these things which I suppose for other people are maybe mundane tasks. It gave me a structure or gave me a routine." P28*
**Personal growth** 68 mentions	Having/raising the dog (17; 25.0%) Training the dog, doing agility, scent work (11; 16.2%) Looking after the dog—general routine (7; 10.3%) The presence of the dog (6; 8.8%) Exercise with the dog (5; 7.4%) Dog shows good behaviour, training skills (4; 5.9%)	*"I've never really sort of connected with anyone or anything up until we got her. […] I would assume people to be in my life short term, there was never any sort of long-term prospect, I was always by myself. When I got her, it was the first time I sort of allowed myself to think or to form a long-term bond, I suppose." P30*
**Positive relations with others** 122 mentions	The presence of the dog (28; 23.0%) Exercise with the dog (24; 19.7%) Talk about the dog (23; 18.9%) Contact with other people while out with dog (12; 9.8%) Interactions on social media (8; 6.6%), Having/raising the dog (7; 5.7%)	*"He's an ex-racing dog, so people like to come up and talk to you about him. I suppose that does give a relationship with others that you wouldn't talk to normally. A common thing with autism is that you don't have many friends. And you don't have many social situations. And I suppose that's giving you a chance of social interaction." P11*
**Purpose in life** 133 mentions	Exercise with the dog (27; 20.3%) Looking after the dog—general routine (24; 18.0%) Feeding, giving water to the dog (21; 15.8%) Having/raising the dog (16; 12.0%) Maintenance of dog (9; 6.8%)	*"The dog gives you something to get up for[…]it's six o'clock, I must feed the dog. Six thirty I must take him out for his walk, you know, seven o'clock, we sit down and watch television together. Eleven o'clock, we go to bed" P11*
**Self-acceptance** 55 mentions	Close dog-owner interactions (15; 27.3%) The presence of the dog (7; 12.7%) Having/raising the dog (6; 10.9%) Tactile interactions initiated by the dog (5; 9.1%) Dog shows good behaviour, training skills (5; 9.1%) Training the dog, doing agility, scent work (4; 7.3%) Looking after the dog—general routine (3; 5.5%)	*"It's an old saying that everyone should have a dog, so they give you unconditional acceptance[…] If I've been out like it, he's so happy to see me [greeting]. You know, you can't be all that bad if you're getting that sort of reaction. You know, you get a friendly reaction, rather than being ignored or so. You know, it does make you feel good." P23*

The numbers next to the subthemes indicate the number of times (and percentage) a subtheme was reported to generate the respective well-being outcome.

**Figure 4:**
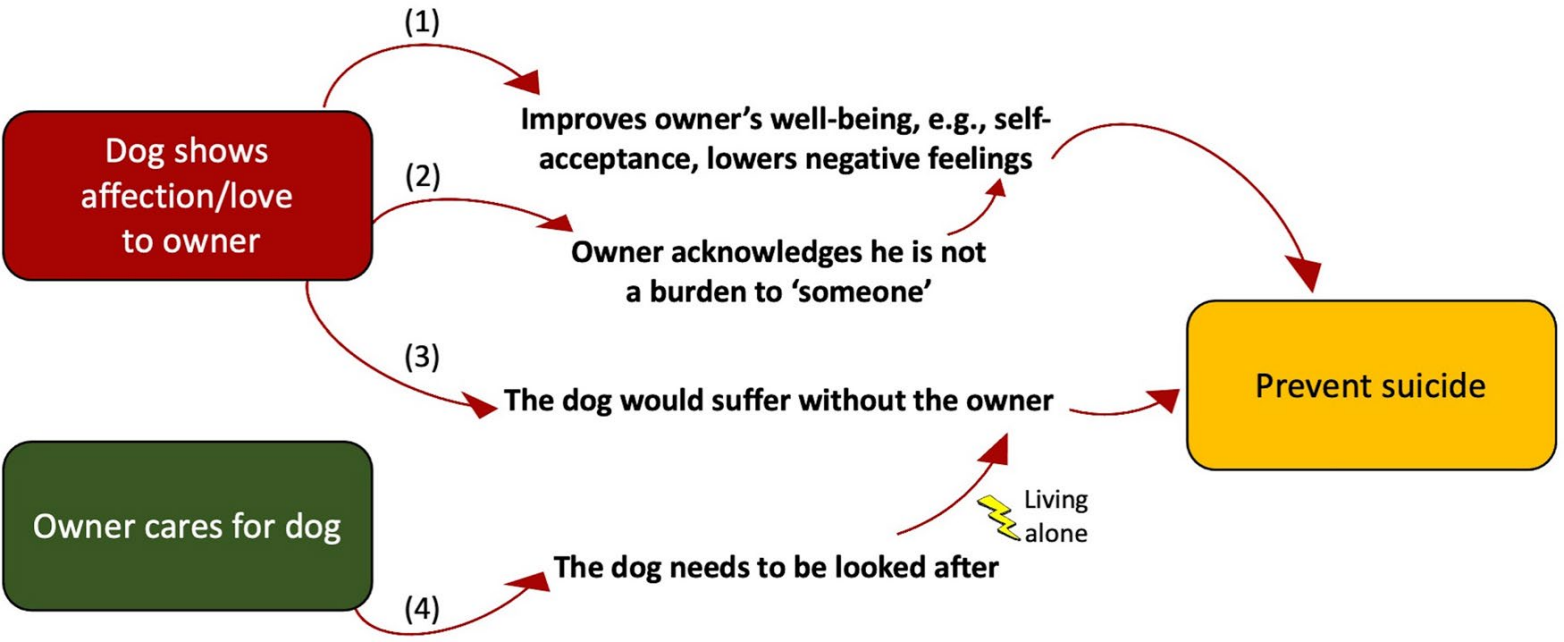
Pathways to suicide prevention through dog ownership amongst autistic adults.

Importantly, six participants (16.7%) reported that their dog was the reason they did not take their own lives. Based on their reports, we describe four pathways to suicide prevention from dog ownership (Fig. 4). Demonstrations of affection/love from the dog to its owner (e.g., dog greets the owner, shows affection) and the care for the dog (e.g., feeding, walking the animal) appear particularly important in this regard, with the fourth pathway, based on living alone with the dog and the prospect of the dog being on its own after the owner’s suicide. Participant 14 describes how his/her dog has prevented him/her from taking his/her own life:

*"He [the dog] is like a protective factor. I have attempted suicide before, and he has helped it stopped happening again. I think ... in the way that I feel that I'm a burden to my family. I don't feel the same about him. Because I think that I meet his needs pretty well. Like that gives me confidence, keeps me going in a way that my family don't."*

Not being able to control their dogs appropriately (e.g., the dog chasing animals, pulling on the lead, poor recall response) hindered various areas of life functioning, and was repeatedly reported by participants (Table 4). Worsening of environmental mastery, personal growth and self-acceptance, for example, were all caused mainly by the dog being out of control. Autonomy was also typically reduced; this was due to the owners' sense of obligation to fulfil their dogs' needs, such as not travelling because the dog couldn’t be left alone. Positive relations with others were negatively affected when participants had unfriendly encounters with other people while out with their dogs or when they didn't want to interact with other individuals, but they still sought contact. Lastly, participants lost purpose in life due to the death of the dog or possibility of dog dying. These are all clearly areas for which these individuals need additional support.

**Table 4:** Activities described to worsen aspects of eudaimonic well-being.

	Most frequent subthemes (≥ 5% mentions)	Sample extract from interviews
**Decrease in**		
**Autonomy** 8 mentions	Sense of obligation to the dog (4; 50.0%) Lack or little control over the dog (2; 25.0%) Maintenance of the dog (1; 12.5%) Looking after the dog—general routine (1; 12.5%)	*"I feel constrained sometimes. So, if I wanted to go on holiday, or be out to visit somewhere else, I would feel that I have to come back for the dogs. And it's not always practical to take them with me, particularly not far on holiday, because I've got three of them." P10*
**Environmental mastery** 5 mentions	Lack of or little control over the dog (2; 40.0%) Sense of obligation to the dog (1; 20.0%) Sensory related (1; 20.0%) Unruly behaviours (1; 20.0%)	*"She just doesn't walk very well on the lead[…]sometimes she'll pull so hard that it really hurts me. […] We've got a kind of Halti head harness now for her, which makes things slightly easier […], but sometimes she can get it off. So it goes from feeling like I've mastered my environment to feeling like incredibly stressed and I just want to go home" P1*
**Personal growth** 3 mentions	Lack of or little control over the dog (2; 66.7%) Death of dog, possibility of dog dying (1; 33.3%)	*"You could actually say personal growth […]. It's like a scolded child syndrome […] So, as a parent, the dog is not listening to me, he is not doing as he is told. That's not acceptable." P24*
**Positive relations with others** 17 mentions	Contact with other people while out with the dog (6; 35.3%) Exercise with the dog (3; 17.6%) Fearful/aggressive behaviours (2; 11.8%) The presence of the dog (2; 11.8%) Meet other people to do dog activity (1; 5.9%) Interactions on social media (1; 5.9%) Tactile interactions initiated by the dog (1; 5.9%) Tactile interactions initiated by the owner (1; 5.9%)	*"I occasionally meet a person with a dog and they've got them on a lead. It's usually a small fluffy thing that travels in a handbag. And they can get very worried because my dogs are off the lead, and some of them got quite aggressive and quite verbally abusive. And that's not a positive relation with others." P35*
**Purpose in life** 1 mention	Death of dog, possibility of dog dying (1; 100%)	*"And then when he passed away, which I suppose you put it into purpose of life. It was one of the most devastating things I've ever had to deal with. And I'm still reeling even yet, like two years later, because he had an impact on me." P26*
**Self-acceptance** 4 mentions	Lack of or little control over the dog (4; 100%)	*"And you feel bad as a teacher, as a trainer, you know[…] I have not trained him well enough. Why is he not coming? Why are you being belligerent when I've asked you to come?" P24*

The numbers next to the subthemes indicate the number of times (and percentage) a subtheme was reported to generate the respective well-being outcome.

### Activities reported to affect life satisfaction

Interacting with the dog through walks, training and physical contact were fundamental to the improvement of participants' life satisfaction, accounting for most of the subthemes of activities reported (Supplementary Table S1). For example, participant 34 said:

*"I think one that, generally speaking, I'd always put in the life satisfaction is taking her for walks. Again, you've got good ones and bad ones. But it adds a nice, almost weekend to the day, you know, we get up, we take the dog for a walk. And then when you finish work, you take the dog for a walk. And it's nice to spend some time with her give her a bit of a run around"*

Besides this, demonstrations of good behaviour by the dog, demonstration of positive affect towards the owner (e.g., following owner, greeting owner) or simply having the dog were also mentioned to improve life satisfaction. Decrease in life satisfaction was not reported.

## Discussion

The 85 dog–human related activities and their well-being outcomes developed from these interviews with 36 autistic adults who own a dog and live in the UK formed a similar framework to the one developed with neurotypical dog owners^42^. This reflects the robustness and generalisability of the original framework. Dog ownership appears to be mostly positive for the well-being of both autistic adults and neurotypicals, and the dog–human related activities they are involved in are similar. A notable feature in the current study was the frequent report of the importance of the dog in preventing suicide (16.7% of the participants), something which had not been mentioned in the research with neurotypicals. Dog-owner interactions (e.g., the presence of the dog, tactile interactions, walking the dog) were a fundamental group of activities linked to improvements in participants' moods/emotions and life functioning (eudaimonic well-being), whereas routine-like activities, such as looking after the dog and simply being an owner, appeared to be particularly linked to the enhancement of eudaimonic well-being. Unwanted behaviours or situations caused by the dog, especially little control over the animal (e.g., dog does not respond to recall) were the leading reason for the worsening of most aspects of well-being; poor dog health/death and obligations to the dog (e.g., maintenance, care) also had a considerable negative impact and we suggest that targeted support should be developed for autistic individuals to help them at these times.

The version of the framework presented here is more diverse in the component dog–human related activities impacting on well-being (n = 85), compared to the original framework (n = 58), with some activities grouped differently. For example, in the previous framework 'exercising with the dog' was an isolated theme, whereas here it became a subtheme of the theme 'shared interactions with the dog'. Such changes were made to increase the comprehensiveness of each theme and to make the framework more accessible and easier to interpret. As expected, novel groups of activities emerged from autistic dog owners, such as not being able to take the dog to places (e.g., to shops), the dog being sick or injured; specific assistance functions (e.g., dog alerts owner of a panic attack); lack of/little control over the dog, etc. Likewise, a few specific activities reported by neurotypical dog owners did not feature here, e.g., cycling, running, sledging and swimming with the dog, cleaning dog's teeth, cooking for the dog, etc. Despite these specific differences, the updated framework covers the themes/subthemes of activities in the lives of both autistic dog owners and neurotypical dog owners, and the impact of these activities on well-being is similar across the two populations. For example, in both frameworks, tactile interactions with the dog improved participants' emotions/moods, training the dog was highly linked to personal growth, looking after the dog was related to purpose in life; dog unwanted behaviours had a negative on various areas of well-being, etc. Thus, the updated framework creates a consistency across different populations of dog owners (at least among neurotypicals and autistic adults) and can be used for testing specific hypotheses relating to the impact of dog ownership on human well-being in future studies.

The lack of studies about dog ownership among autistic adults and the need for improving their mental health support^56^ triggered the investigations of the current study. Dog-owner interactions (e.g., tactile interactions, dog's presence, walking the dog, being greeted by the animal) were the main activities reported to improve emotions/moods in autistic adults (and previously among neurotypicals^42^). This might relate at a physiological level to increases in oxytocin, dopamine, endorphins, prolactin and reductions in cortisol, epinephrine, norepinephrine, which are reported to occur from interacting with dogs^57–63^. The activities reported in the current study provide further insight to help explain, at a behavioural level, why acquiring a pet dog has the potential to reduce anxiety in autistic children^36^ and lower parenting stress in families with autistic children^37,38^, and even why the introduction of a service dog in families with autistic children was accompanied by a decrease in children's awakening cortisol level^64^ and increase in child's safety and calmness^65^. The dog may be seen as a form of complex intervention with multiple effects depending on context.

Life satisfaction was rarely (1.7% of all the mentions) linked to dog–human related activities in this study, but the well-being outcome was only ever positive. The main activity described to enhance life satisfaction, dog walking, has been associated with various psychological and physical benefits in humans^66–69^, which perhaps suggests a range of ways it may have the potential to improve life satisfaction. Other authors have also found that having a dog^20^ and spending time with dogs^70,71^ are linked to higher life satisfaction, in line with our results.

The dog's presence and caring for the dog (e.g., walks) was central to increasing participants' sense of autonomy and environmental mastery, mainly by acting as a 'tangible support' (someone to aid in more practical, instrumental tasks^72^), helping owners to perform daily life activities (e.g., shopping, walking, working) and leave their houses. Although few studies have assessed the impact of dogs on human autonomy (e.g. ^20,42,73^,), animal-assisted interventions with the elderly^74–76^ and pet ownership among the elderly^77,78^ seem to improve or prevent deterioration in the performance of daily life activities, an important aspect of environmental mastery. Thus, dog's social support^79–85^ and caring for them seem to have a consequential role in enhancing human independence and control over the environment.

Participants described that having a dog and training their animals gave them a sense of achievement, essential to their personal growth, as reported also by Barcelos et al*.*^42^. Results from previous studies that assessed the impact of dog training on human well-being, mostly with offenders (e.g. ^86–88^,), indicate that the effect of training on personal growth is not restricted to dog owners. In a study with prisoners, who prepared rescue dogs for rehoming through an 8-week positive reinforcement training program, participants said that training dogs improved their skills, gave them a sense of development, achievement, and made them feel proud of themselves^89^. Self-improvements, such as patience, impulse control, parenting skills, assertiveness have also been reported by soldiers with post-traumatic stress disorder involved in dog training programs^90^. Thus, besides the inherent benefits of training to animals, dog training might be useful to enhance personal growth in adults who need or want to improve this area of functioning.

The effect of dogs as social catalysts and lubricants is well referenced in the literature^85,91–93^ and so it was not surprising that, as with neurotypicals^42^, the dog's presence and use as a topic for conversation was central to improving participants' positive relationship with others (e.g., facilitating interactions with strangers, strengthening their relationships in the neighbourhood). Several studies have indicated that the presence of a dog^94–98^ or other animals^99,100^, in therapeutic and educational contexts, is beneficial to autistic children's social interactions and the regular presence of a dog in the lives of those who struggle or want to improve their social relations could be beneficial.

The routine of looking after the dog (e.g., walking, feeding), was important to improving the purpose in life of autistic adults (as was found with neurotypicals^42^), giving additional meaning to their lives. The importance of this relationship is supported by several other qualitative studies with dog owners (e.g. ^26,31,101,102^,). Thus, caring for a dog also seems to have the potential to be used therapeutically to enhance purpose in life in adults with mental health issues, such as depression, in which lack of interest/pleasure in activities of the day is a common symptom^6^. In fact, Pereira and Fonte^103^ found that depression levels dropped significantly more, after eight and twelve weeks, in severely depressed individuals who acquired a pet compared to those who did not acquire one, and the authors believe that looking after the animal played a key role in these changes.

Finally, self-acceptance in autistic adults was reported to be boosted by interactions initiated by the dog (e.g., greeting the owner, showing affection) and its mere presence. Interestingly, in a cross-sectional study with 217 adults, those who owned a pet reported significantly higher self-esteem than non-owners, and pets were reported to provide as much social support as the participants' parents or siblings^104^. This may be particularly important as lower internal and external acceptance in autism has been associated with mental health problems^105^.

However, it is important to appreciate that there were also negative impacts (e.g., due to dog behaviour problems, dog's death/sickness), and those seeking to use dogs to help individual dog-lovers with autism should consider how to mitigate against these. Parents of autistic children have also reported that the child can be irritated or bothered by their pet^41^, can dislike their dog because of its behaviour, or have sensory issues with the dog (e.g., jumping, barking, licking^40^). As identified elsewhere in other populations^42,106–109^, dog's unwanted behaviour was frequently reported to worsen participants' hedonic and eudaimonic well-being. Even during the COVID-19 pandemic, when people may be expected to benefit greatly from the social support of their animals (e.g., loneliness protection^22,23^), dogs' undesirable behaviours impact negatively on human well-being^108^. These may be quite common, with 'human focused' issues linked to their pets, such as barking, disrupting owner activities and attention seeking, reported by 23% of the 2254 US pet owners involved in Applebaum's et al*.*'s^108^ study. Unwanted dog behaviours are not only detrimental to owner well-being but also to the dog-owner relationship^107^, being one of the leading causes of relinquishment^110,111^. Finally, dog's poor health (e.g., death, sickness, injury) and a sense of obligation to the dog (e.g., failing to/having to care for it) were also reported to negatively impact participant well-being; an effect which has been described in other investigations^42,112–116^. Thus, caregiving burden and a potential grief should be carefully considered before the acquisition of a dog by vulnerable individuals.

The high proportion of participants who said that their dog was the reason they did not take their own lives (16.7%) was unexpected. This figure is probably the product of both the high level of suicidality in autistic adults^11–14^ as well as the potential importance of the dog in mitigating this risk. Pelton et al*.*^117^ identified three important risk factors for suicide among autistic adults: stronger feelings of thwarted belonging (lack of reciprocal relationships with others), stronger feelings of perceived burden to others (that others would be better off without them) and lifetime trauma, the two first being part of the widely cited Interpersonal Theory of Suicide^118^. The potential role of dog ownership on mitigating traumatic life events was not a focus of this study, but a potential reduction in thwarted belonging and burdensomeness seem to be revealed from two elements (Fig. 4):

1. looking after the dog, which makes owners feel needed (by the dog) and their feelings reciprocated, leading them to reflect on the suffering the animal would face if they were to commit suicide.
2. demonstrations of affection from the dog to its owner, which make owners feel they are not burden to the dog, but rather that they are important and their dog would be worse off without them.

These two elements also highlight a “responsibility” variable that seems to be a strongly protective factor against suicide in people living with dogs—the owner is the main person responsible for the dog; thus, he/she cannot leave the dog behind, particularly when he/she lives alone (usually a risk factor for suicide^119,120^).

In an editorial, Batty and Bell^109^ reported that no relationship was found between pet ownership (or dog ownership) and suicide in a prospective investigation that included 47 suicide deaths. However, it was not known to what extent the owners were involved in the care of the pet, a limitation acknowledged by the authors, nor if the pet showed affection towards them, both potentially fundamental elements to the value of dogs in preventing suicide, in the pathways identified here. This also serves to highlight the problems with simply considering dog ownership as a simple or homogenous variable, a fallacy emphasised by the diversity of the framework described here. Indeed, Love^122^ conducted a qualitative study with 71 adult pet owners who had had suicidal thoughts or behaviours and identified three pet-related protective factors against suicide: comfort provided by the pet (e.g., emotional support), distraction from suicidal thoughts (e.g., pet seeks attention and distracts owner) and a reason to live (e.g., obligation to care for the pet). These factors match very well the two main elements of suicide prevention among autistic dog owners identified here (affection shown by the dog and care for the animal). Nonetheless, Love^122^ also pointed out two risk factors for suicide, i.e., pets' behaviour problems and pets' health problems. Although uncommon, these risk factors were the same two negative themes identified in the current study which worsened both hedonic and eudaimonic well-being. Such consistency in both risk factors and protective factors between the two populations of pet owners highlights the real importance and potential of investigating further pathways to suicide prevention through dog ownership.

This is an exploratory study with a limited number of participants restricted to those able to engage with the research methodology (qualitative interviews^123^). It therefore does not cover the full spectrum of the condition. The mechanisms found here to explain how exactly dog ownership affects human well-being should be investigated further in large quantitative studies, as this qualitative work provides the initial insights into the topic. However, saturation of our data, demographic balance within our sample and extensive geographical variation among participants are strengths of this study. Another potential issue, common in investigations with pet owners, was the predominant participation of individuals who were happy about their dogs and the relationship they have, which may have overshadowed the negative impact dogs can have on well-being. Nevertheless, saturation was reached within all themes, including those predominantly negative to well-being (e.g., dog unwanted behaviours/situations; dog's health), and the authors clearly emphasised the potential negative impact dogs can have on human well-being to participants. The identification of these areas is crucial if we wish to maximise the benefits while minimising the risks associated with ownership in this population. Finally, improvements in life satisfaction were infrequently mentioned by the participants and this could be related to our methodology (i.e., participants may have struggled to think about specific dog–human related activities in relation to life satisfaction; they may have focused more on affect, as both life satisfaction and affect were approached together). Alternatively, it may reflect genuinely little impact on life satisfaction, although quantitative studies indicate that interacting/having a dog can be beneficial to life satisfaction^20,70,71^.

The variability of dog–human related activities and well-being outcomes reported here by autistic adults and by neurotypicals in Barcelos et al*.*^42^ reinforces the heterogeneity of dog ownership^124,125^ and the need to recognise and address this in future investigations. Frequently, studies have simply compared the well-being of dog owners versus non-owners, with little or no consideration of the full content of the relationship; apparently hoping to show evidence of a general 'pet effect' (i.e., pet's ability to improve human physical and psychological well-being^126^). It is thus not surprising that there is growing evidence of a lack of consistent effect^127^. By considering specific aspects of dog ownership as revealed in this study (e.g., dog's health; level of shared interactions with the dog; dog unwanted behaviours; level of involvement in the care of the dog) and acknowledging both the positive and negative impact dog ownership has on well-being^42,108,128^, researchers can test more specific hypotheses. In this way it is to be hoped we can build a more accurate picture of the typical aspects of ownership which may be beneficial to human well-being and thus elucidate the true diversity of underlying mechanisms. Only then can we hope to build a rational evidence base for maximising the benefits while minimising the risks of dog (or more general animal) related activities.

Critically, our study suggests dog ownership prevented suicide by several autistic adults in this study. As a crude approximation to illustrate the significance of this point (but not the actual numbers involved); let us consider a simple scaling up of our data. If we assume 1% of the 52 million adults in the UK have autism and that dog ownership among this population is as popular as other adults at 26%, then dog ownership would be responsible for preventing around 22 thousand suicides among 135 thousand autistic adults in the UK alone. We do not claim this is the actual figure, but do believe this rough exercise highlights the importance of this. There is a need for research on pet ownership and suicide^121^, particularly in more vulnerable groups like autistic adults. With the aid of future longitudinal and large-scale investigations, the mechanism for suicide prevention identified in this qualitative investigation could be confirmed in a more representative population, and strategies of suicide prevention based on dog–human interactions recommended with greater scientific evidence. In addition, there is enormous potential to use the framework for testing specific hypotheses relating to the impact of dog–human related activities within animal-assisted activities and the wider dog-owning population. Two common major problems with studies of animal-assisted interventions are the lack of a strong explanatory theory for the potential effects on humans and the lack of standardized intervention procedures^85^. Is it about touching the dog, gazing at the dog, the dog’s presence, its role as a social catalyst, increased activity (e.g., walking), or the experience of training an animal? Interventional variables are commonly mixed and not quantified (e.g. ^74,129–133^,), making it hard to identify the root of the change and to replicate studies. The framework presented here provides the basis for such explanatory theory and standardisation.

## Conclusion

Our findings indicate that when an autistic adult chooses to have a dog, the impact is generally beneficial, rather than negative, and that it has the potential of saving the lives of those considering suicide, particularly when the dog demonstrates affection towards its owner and the owner is responsible for looking after the animal. Other benefits go far beyond suicide prevention, dogs can help autistic adults perform simple everyday tasks, such as the purchase of products in shops, and can also be very helpful to keep a routine in their lives and facilitate conversations with other individuals, which, otherwise, without a dog, could be extremely challenging for many autistic adults. Nevertheless, before acquiring a dog, it is also important to consider the negative impact of specific dog–human related activities both on autistic and neurotypical adults, to avoid disappointment and other negative effects and ultimately the relinquishment of the dog. Finally, the updated framework described here can guide the formulation of hypotheses in future studies about dog–human interaction and human well-being. Although mild variations in the framework are expected to occur across different samples, its core themes and well-being outcomes appear reasonably robust.

## Supplementary Information

Supplementary Information.

## Data Availability

Data used for analysis are included in this published article (and its Supplementary Information file).
