# Author Correction: Understanding the impact of dog ownership on autistic adults: implications for mental health and suicide prevention

**DOI:** 10.1038/s41598-021-04063-4

**Published:** 2021-12-21

**Authors:** Ana Maria Barcelos, Niko Kargas, Chris Packham, Daniel S. Mills

**Affiliations:** 1 School of Life Sciences, University of Lincoln, Lincoln, UK; 2 School of Psychology, University of Lincoln, Lincoln, UK

Correction to: *Scientific Reports* 10.1038/s41598-021-02504-8, published online 08 December 2021

The original version of this Article contained an error in the Discussion section, where.

"If we assume 1% of the 52 million adults in the UK have autism and that dog ownership among this population is as popular as other adults at 26%, then dog ownership would be responsible for preventing around 135 thousand suicides among autistic adults in the UK alone."

now reads:

"If we assume 1% of the 52 million adults in the UK have autism and that dog ownership among this population is as popular as other adults at 26%, then dog ownership would be responsible for preventing around 22 thousand suicides among 135 thousand autistic adults in the UK alone."

The original Article has been corrected.

