# Supplementary material for: Understanding the impact of dog ownership on autistic adults: implications for mental health and suicide prevention
Source: Sci Rep. 2021 Dec 8;11:23655. doi: 10.1038/s41598-021-02504-8 (PMC8655007; doi:10.1038/s41598-021-02504-8)
Supplement: Supplementary file 1 — Supplementary Information. [file 41598_2021_2504_MOESM1_ESM.pdf]

Understanding the impact of dog ownership on autistic adults: implications for mental health and suicide prevention

Barcelos, Ana Maria[1]; Kargas, Niko[2]; Packham, Chris[1]; Mills, Daniel S.[1]

[1]School of Life Sciences, University of Lincoln, Lincoln, United Kingdom
[2]School of Psychology, University of Lincoln, Lincoln, United Kingdom

Table S1. Activities described to impact on life satisfaction

**Well-being outcome**	**Most frequent subthemes (≥5% mentions) and the most common activity within that subtheme**
Increase in life satisfaction (21 mentions)	°**Exercise with the dog (4; 19.0%)**<br>°**Dog shows good behaviour, training skills (3; 14.3%)**<br>°**Training the dog, doing agility, scent work (3; 14.3%)**<br>°**Tactile interactions initiated by the owner or unclear (3; 14.3%)**<br>°**Having/raising the dog (2; 9.5%)**<br>°**Close dog-owner interactions (2; 9.5%)**<br>°**Tactile interactions initiated by the dog (2; 9.5%)**
Decrease in life satisfaction (0 mentions)	No activity has been reported to decrease life satisfaction

Table S2. Characteristics of the 36 participants and their dogs

Category	n	%	Category	n	%
Sex			Anxiety (GAD-7)		
Female	18	50.0%	*No symptoms*	1	2.8%
Male	18	50.0%	*Minimal*	2	5.6%
Age (in years)			*Mild*	15	41.7%
18-24	5	13.9%	*Moderate*	7	19.4%
25-34	14	38.9%	*Severe*	11	30.6%
35-44	7	19.4%	Number of dogs owned		
45-54	6	16.7%	*1*	23	63.9%
55-54	3	8.3%	*2*	8	22.2%
65-74	1	2.8%	*3 or more*	5	13.9%
Location in the UK			Sex of the dog*		
England			*Female*	20	55.6%
East Midlands	4	11.1%	*Male*	23	63.9%
East of England	2	5.6%	Age of the dog*		
Greater London	5	13.9%	*Below 12 months*	3	8.3%
North East England	1	2.8%	*1-3 years*	16	44.4%
North West England	5	13.9%	*4-6 years*	12	33.3%
South East England	5	13.9%	*7-9 years*	13	36.1%
South West England	6	16.7%	*10 or more years*	5	13.9%
West Midlands	2	5.6%	Length of dog ownership*		
Scotland	3	8.3%	*Below 12 months*	4	11.1%
Wales	3	8.3%	*1-3 years*	19	52.8%
Expertise in dog-related field			*4-6 years*	14	38.9%
Yes, e.g., training, behaviour	6	16.7%	*7-9 years*	7	19.4%
No	30	83.3%	*10 or more years*	4	11.1%
Depression (PHQ-9):			Reason for acquiring dog*		
No symptoms	0	0.0%	*Autism service dog*	6	16.7%
Minimal	7	19.4%	*Other: pet, company, sport*	34	94.4%
Mild	12	33.3%	Size of the dog*		
Moderate	9	25.0%	*Small*	8	22.2%
Moderately severe	5	13.9%	*Medium*	24	66.7%
Severe	3	8.3%	*Large*	9	25.0%

* % exceeds 100% in these categories as the 'n' is the number of participants, not dogs.

Dog human related activities (themes, subthemes and activities)	HEDONIC WELL-BEING								EUDAIMONIC WELL-BEING												LS ↑ LS	Number of mentions
	Increase in				Decrease in				Increase in						Decrease in							
	NH	NL	PH	PL	NH	NL	PH	PL	Au	En	Pe	Po	Pu	Se	Au	En	Pe	Po	Pu	Se	LS	
I.Inevitable features of dog ownership																						
a. Having/raising the dog																						
1.Having the dog	0.0%	0.0%	3.0%	1.6%	2.9%	11.8%	0.0%	0.0%	19.1%	7.4%	19.1%	5.7%	12.0%	9.1%	0.0%	0.0%	0.0%	0.0%	0.0%	0.0%	4.8%	72
2.Raising the dog	0.0%	0.0%	1.2%	0.5%	0.0%	0.0%	0.0%	0.0%	0.0%	0.0%	5.9%	0.0%	0.0%	1.8%	0.0%	0.0%	0.0%	0.0%	0.0%	0.0%	4.8%	9
b. Maintenance of dog, e.g., costs, trips to the vet, food/object purchases																						
3.General costs of keeping dog, e.g.,vet, insurance, food, etc	2.9%	0.0%	0.0%	0.0%	0.0%	0.0%	0.0%	0.0%	0.0%	0.0%	0.0%	0.0%	0.0%	0.0%	12.5%	0.0%	0.0%	0.0%	0.0%	0.0%	0.0%	6
4.Take dog to the vet, plan vet visits	2.9%	0.0%	0.0%	0.5%	0.0%	0.0%	0.0%	0.0%	2.1%	1.1%	1.5%	1.6%	0.0%	0.0%	0.0%	0.0%	0.0%	0.0%	0.0%	0.0%	0.0%	11
5.Buying or planing to buy things for the dog, e.g., food, clothes, toys, treats	1.1%	0.0%	1.2%	1.1%	0.0%	0.0%	0.0%	0.0%	4.3%	2.1%	0.0%	0.0%	4.5%	0.0%	0.0%	0.0%	0.0%	0.0%	0.0%	0.0%	0.0%	16
6.Find a better house, earn more money, stop drinking for the dog	0.0%	0.0%	0.0%	0.0%	0.0%	0.0%	0.0%	0.0%	0.0%	0.0%	1.5%	0.0%	2.3%	0.0%	0.0%	0.0%	0.0%	0.0%	0.0%	0.0%	0.0%	4
c.7.Not being able to take dog to places, e.g., restrictions in shops, hotels	1.7%	3.2%	0.0%	0.0%	0.0%	0.0%	0.0%	0.0%	2.1%	0.0%	0.0%	0.0%	0.0%	0.0%	0.0%	0.0%	0.0%	0.0%	0.0%	0.0%	0.0%	5
II.Dog's health																						
a.8.Dog sick or injured, e.g.the situation, treatment	8.6%	3.2%	0.0%	0.0%	0.0%	0.0%	0.0%	0.0%	2.1%	0.0%	0.0%	0.0%	0.8%	1.8%	0.0%	0.0%	0.0%	0.0%	0.0%	0.0%	0.0%	19
b.9.Old dog, approaching the end of dog's life	1.1%	0.0%	0.0%	0.0%	0.0%	0.0%	0.0%	0.0%	0.0%	0.0%	0.0%	0.0%	0.0%	0.0%	0.0%	0.0%	0.0%	0.0%	0.0%	0.0%	0.0%	2
c.10.Death of dog (e.g., euthanasia), possibility of dog dying	2.9%	12.9%	0.0%	0.0%	0.0%	0.0%	0.0%	0.0%	0.0%	0.0%	0.0%	0.0%	0.0%	0.0%	0.0%	0.0%	33.3%	0.0%	100.0%	0.0%	0.0%	11
III.Shared interactions with the dog																						
a. The presence of the dog - inside and outside the house																						
11.Dog's presence - general	0.0%	0.0%	1.8%	2.2%	5.7%	11.8%	0.0%	0.0%	4.3%	3.2%	1.5%	0.0%	0.8%	1.8%	0.0%	0.0%	0.0%	0.0%	0.0%	0.0%	4.8%	24
12.Dog's presence in the house	0.0%	0.0%	0.0%	2.7%	4.3%	2.9%	0.0%	0.0%	2.1%	2.1%	0.0%	1.6%	0.8%	0.0%	0.0%	0.0%	0.0%	0.0%	0.0%	0.0%	0.0%	15
13.Dog's presence while meeting people	0.0%	0.0%	1.8%	1.1%	4.3%	2.9%	0.0%	0.0%	0.0%	0.0%	0.0%	6.6%	0.0%	3.6%	0.0%	0.0%	0.0%	0.0%	0.0%	0.0%	0.0%	19
14.Dog's presence while out, e.g. shopping, walking, travelling, at work	0.0%	0.0%	7.2%	4.9%	11.4%	8.8%	0.0%	0.0%	19.1%	10.5%	7.4%	14.8%	0.0%	7.3%	0.0%	0.0%	0.0%	11.8%	0.0%	0.0%	0.0%	80
b.Reduced dog interaction, e.g., not having the dog outside, receiving little affection from the dog																						
15.Not having the dog while out	2.3%	9.7%	0.0%	0.0%	0.0%	0.0%	0.0%	0.0%	0.0%	0.0%	0.0%	0.0%	0.0%	0.0%	0.0%	0.0%	0.0%	0.0%	0.0%	0.0%	0.0%	7
16.Lack of or little affection from the dog to human	0.6%	0.0%	0.0%	0.0%	0.0%	0.0%	0.0%	0.0%	0.0%	0.0%	0.0%	0.0%	0.0%	0.0%	0.0%	0.0%	0.0%	0.0%	0.0%	0.0%	0.0%	1
c.Exercise with the dog, e.g., walking, hiking																						0
17.Hiking	0.0%	0.0%	0.0%	0.5%	1.4%	0.0%	0.0%	0.0%	0.0%	0.0%	0.0%	0.0%	0.0%	0.0%	0.0%	0.0%	0.0%	0.0%	0.0%	0.0%	0.0%	2
18.Walking	1.1%	3.2%	7.8%	9.3%	4.3%	5.9%	0.0%	0.0%	17.0%	24.2%	7.4%	19.7%	20.3%	1.8%	0.0%	0.0%	0.0%	17.6%	0.0%	0.0%	19.0%	133
d.Watching dog's behaviour, e.g., happy/unhappy dog																						0
19.See dog happy, having fun	0.0%	0.0%	8.4%	2.7%	0.0%	0.0%	0.0%	0.0%	0.0%	0.0%	0.0%	0.0%	0.0%	0.0%	0.0%	0.0%	0.0%	0.0%	0.0%	0.0%	4.8%	20
20.See dog unhappy, upset	0.6%	3.2%	0.0%	0.0%	0.0%	0.0%	0.0%	0.0%	0.0%	0.0%	0.0%	0.0%	0.0%	0.0%	0.0%	0.0%	0.0%	0.0%	0.0%	0.0%	0.0%	2
e.Specific assistance functions, e.g., dog alerts owner of a panic attack.																						
21.Dog alerts owner of a panic attack, high anxiety, PoTS	0.0%	0.0%	0.0%	1.1%	4.3%	0.0%	0.0%	0.0%	0.0%	0.0%	0.0%	0.0%	0.0%	0.0%	0.0%	0.0%	0.0%	0.0%	0.0%	0.0%	0.0%	5
22.Dog stands in front of owner to keep people more distant	0.0%	0.0%	0.0%	0.5%	1.4%	0.0%	0.0%	0.0%	0.0%	0.0%	0.0%	0.0%	0.0%	0.0%	0.0%	0.0%	0.0%	0.0%	0.0%	0.0%	0.0%	2
23.Owner puts dog on the chest, carry, to receive pressure	0.0%	0.0%	0.0%	0.5%	1.4%	0.0%	0.0%	0.0%	0.0%	0.0%	0.0%	0.0%	0.0%	0.0%	0.0%	0.0%	0.0%	0.0%	0.0%	0.0%	0.0%	2
f.Close dog-owner interactions, e.g., dog greets owner, talking to the dog																						
24.Dog asking to be petted, e.g.,belly up	0.6%	0.0%	0.6%	2.2%	5.7%	0.0%	0.0%	0.0%	0.0%	0.0%	0.0%	0.0%	0.0%	0.0%	0.0%	0.0%	0.0%	0.0%	0.0%	0.0%	0.0%	10
25.Dog follows the owner or seeks owner for refuge/protection	1.1%	0.0%	1.2%	1.6%	0.0%	0.0%	0.0%	0.0%	0.0%	0.0%	0.0%	0.0%	0.8%	7.3%	0.0%	0.0%	0.0%	0.0%	0.0%	0.0%	4.8%	13
26.Dog looks at owner	0.0%	0.0%	0.0%	0.0%	1.4%	0.0%	0.0%	0.0%	0.0%	1.1%	0.0%	0.0%	0.0%	1.8%	0.0%	0.0%	0.0%	0.0%	0.0%	0.0%	0.0%	3
27.Dog shows affection, love towards owner - general, non-specified	0.6%	0.0%	0.6%	1.6%	1.4%	8.8%	0.0%	0.0%	0.0%	0.0%	1.5%	0.0%	0.8%	9.1%	0.0%	0.0%	0.0%	0.0%	0.0%	0.0%	0.0%	16
28.Dog wakes owner up	0.6%	0.0%	0.6%	0.0%	0.0%	0.0%	0.0%	0.0%	0.0%	2.1%	0.0%	0.0%	1.5%	0.0%	0.0%	0.0%	0.0%	0.0%	0.0%	0.0%	0.0%	6
29.Greeting the owner	0.6%	0.0%	5.4%	3.3%	2.9%	2.9%	0.0%	0.0%	0.0%	1.1%	0.0%	0.0%	1.5%	9.1%	0.0%	0.0%	0.0%	0.0%	0.0%	0.0%	4.8%	28
30.Taking picture,videos of own dog	0.0%	0.0%	1.2%	0.0%	0.0%	0.0%	0.0%	0.0%	0.0%	0.0%	0.0%	1.6%	0.0%	0.0%	0.0%	0.0%	0.0%	0.0%	0.0%	0.0%	0.0%	4
31.Talking to the dog	0.0%	0.0%	0.6%	1.1%	0.0%	0.0%	0.0%	0.0%	0.0%	0.0%	0.0%	0.8%	0.0%	0.0%	0.0%	0.0%	0.0%	0.0%	0.0%	0.0%	0.0%	4
g.Tactile interactions (or very close) initiated by the dog, e.g.,dog lies on the owner																						0
32.Dog licks, kisses the owner	0.6%	0.0%	2.4%	2.2%	2.9%	11.8%	0.0%	0.0%	0.0%	2.1%	0.0%	0.0%	0.0%	3.6%	0.0%	0.0%	0.0%	0.0%	0.0%	0.0%	0.0%	19
33.Dog sits, lies on the owner or cuddles with owner	0.6%	0.0%	4.2%	12.6%	14.3%	5.9%	0.0%	0.0%	0.0%	3.2%	1.5%	0.0%	0.0%	3.6%	0.0%	0.0%	0.0%	0.0%	0.0%	0.0%	4.8%	50
34.Dog sits, lies next to the owner	0.0%	0.0%	1.8%	4.9%	4.3%	2.9%	0.0%	0.0%	0.0%	0.0%	0.0%	0.0%	0.8%	0.0%	0.0%	0.0%	0.0%	0.0%	0.0%	0.0%	4.8%	18
35.Dog sleeps next to owner, e.g., in bed	0.6%	3.2%	1.2%	2.2%	0.0%	0.0%	0.0%	0.0%	0.0%	0.0%	0.0%	0.0%	0.0%	0.0%	0.0%	0.0%	0.0%	5.9%	0.0%	0.0%	0.0%	9
36.Dog touches the owner with paw or face, e.g., nudges with nose	0.6%	0.0%	0.6%	2.7%	5.7%	5.9%	0.0%	0.0%	0.0%	1.1%	0.0%	0.0%	0.0%	1.8%	0.0%	0.0%	0.0%	0.0%	0.0%	0.0%	0.0%	15
h.Tactile interactions (or very close) initiated by the owner or unclear, e.g., petting the dog																						0
37.Being next to the dog	0.6%	0.0%	0.0%	1.1%	1.4%	2.9%	0.0%	0.0%	0.0%	0.0%	0.0%	0.0%	0.0%	1.8%	0.0%	0.0%	0.0%	5.9%	0.0%	0.0%	4.8%	8
38.Cuddling, stroking, petting the dog	0.0%	0.0%	3.0%	8.8%	7.1%	2.9%	0.0%	0.0%	0.0%	1.1%	0.0%	0.0%	1.5%	1.8%	0.0%	0.0%	0.0%	0.0%	0.0%	0.0%	4.8%	32
39.Grooming related, e.g., bathing, brushing,trimming	0.6%	3.2%	0.6%	2.2%	1.4%	0.0%	0.0%	0.0%	0.0%	1.1%	1.5%	0.0%	2.3%	0.0%	0.0%	0.0%	0.0%	0.0%	0.0%	0.0%	0.0%	13
40.Physical contact with dog - general	0.0%	0.0%	0.0%	1.1%	1.4%	2.9%	0.0%	0.0%	0.0%	0.0%	0.0%	0.0%	0.0%	0.0%	0.0%	0.0%	0.0%	0.0%	0.0%	0.0%	4.8%	5
41.Watching tv together	0.0%	0.0%	0.0%	1.1%	0.0%	0.0%	0.0%	0.0%	0.0%	0.0%	0.0%	0.0%	0.0%	0.0%	0.0%	0.0%	0.0%	0.0%	0.0%	0.0%	0.0%	2
IV.Educational impacts																						
a.42.Learning dog related things, e.g., dog psychology, new approaches	0.0%	0.0%	0.0%	0.0%	0.0%	0.0%	0.0%	0.0%	0.0%	0.0%	2.9%	0.0%	0.0%	0.0%	0.0%	0.0%	0.0%	0.0%	0.0%	0.0%	0.0%	2
b.43.Dog shows good behaviour, training skills	0.0%	0.0%	7.8%	3.3%	0.0%	0.0%	0.0%	0.0%	0.0%	0.0%	5.9%	0.0%	0.0%	9.1%	0.0%	0.0%	0.0%	0.0%	0.0%	0.0%	14.3%	31
c.Training the dog, doing agility, scent work																						
44.Specific activities - dog agility, scent work	0.0%	0.0%	3.0%	1.1%	0.0%	0.0%	0.0%	0.0%	0.0%	0.0%	0.0%	0.8%	0.0%	1.8%	0.0%	0.0%	0.0%	0.0%	0.0%	0.0%	0.0%	9
45.Training the dog, e.g., obedience, socialisation	1.1%	0.0%	7.2%	1.6%	2.9%	0.0%	0.0%	0.0%	4.3%	1.1%	16.2%	1.6%	2.3%	5.5%	0.0%	0.0%	0.0%	0.0%	0.0%	0.0%	14.3%	44
d.46.Punishing the dog physically or verbally	1.1%	3.2%	0.0%	0.0%	0.0%	0.0%	50.0%	0.0%	0.0%	0.0%	0.0%	0.0%	0.0%	0.0%	0.0%	0.0%	0.0%	0.0%	0.0%	0.0%	0.0%	4
V.Looking after the dog																						0
a.47.Cleaning after the dog, e.g. pick up dog poo, clean soiled area	2.9%	0.0%	0.0%	0.0%	0.0%	0.0%	0.0%	0.0%	2.1%	0.0%	0.0%	0.0%	2.3%	0.0%	0.0%	0.0%	0.0%	0.0%	0.0%	0.0%	0.0%	9
b.48.Stimulating the dog intellectually, enrichments	0.0%	3.2%	0.0%	0.0%	0.0%	0.0%	0.0%	0.0%	0.0%	0.0%	1.5%	0.0%	0.8%	0.0%	0.0%	0.0%	0.0%	0.0%	0.0%	0.0%	0.0%	3
c.49.Feeding, giving water to the dog	0.0%	3.2%	3.6%	3.8%	0.0%	0.0%	0.0%	0.0%	2.1%	12.6%	2.9%	0.8%	15.8%	1.8%	0.0%	0.0%	0.0%	0.0%	0.0%	0.0%	0.0%	52
d.50.Looking after the dog - general routine	0.0%	0.0%	3.0%	2.2%	0.0%	0.0%	0.0%	0.0%	10.6%	12.6%	10.3%	0.8%	18.0%	5.5%	12.5%	0.0%	0.0%	0.0%	0.0%	0.0%	0.0%	62
e.51.Taking dog to the toilet, letting dog out	0.0%	0.0%	0.6%	0.5%	0.0%	0.0%	0.0%	0.0%	0.0%	4.2%	0.0%	0.0%	3.8%	0.0%	0.0%	0.0%	0.0%	0.0%	0.0%	0.0%	0.0%	11
f.Sense of obligation to the dog, e.g., failing to walk the dog, having to give company to it																						0
52.Failure to fulfill dog's needs, e.g., leave the dog alone, do not walk the dog	1.7%	3.2%	0.0%	0.0%	0.0%	0.0%	0.0%	0.0%	0.0%	0.0%	0.0%	0.0%	0.0%	0.0%	0.0%	20.0%	0.0%	0.0%	0.0%	0.0%	0.0%	5
53.Having to fulfill dog's needs, e.g., give company, walk, food	1.1%	0.0%	0.0%	0.0%	0.0%	2.9%	0.0%	0.0%	8.5%	1.1%	0.0%	0.0%	1.5%	0.0%	50.0%	0.0%	0.0%	0.0%	0.0%	0.0%	0.0%	14
VI.Play related																						0
a.Dog playing with owner, another dog or object																						
54.Dog playing with object	0.6%	0.0%	1.2%	0.0%	0.0%	0.0%	0.0%	33.3%	0.0%	0.0%	0.0%	0.0%	0.0%	0.0%	0.0%	0.0%	0.0%	0.0%	0.0%	0.0%	0.0%	4
55.Dog playing with other dogs	0.0%	6.5%	1.2%	0.0%	0.0%	0.0%	0.0%	0.0%	0.0%	0.0%	0.0%	0.8%	0.0%	0.0%	0.0%	0.0%	0.0%	0.0%	0.0%	0.0%	0.0%	5
56.Dog playing with owner	0.0%	0.0%	6.6%	2.2%	2.9%	0.0%	0.0%	0.0%	0.0%	4.2%	0.0%	0.0%	2.3%	0.0%	0.0%	0.0%	0.0%	0.0%	0.0%	0.0%	0.0%	24
VII.Social interactions with others																						0
a.57.Contact with other people while out with dog, e.g. other dog owners	3.4%	0.0%	0.6%	1.1%	0.0%	0.0%	0.0%	0.0%	0.0%	0.0%	4.4%	9.8%	0.0%	3.6%	0.0%	0.0%	0.0%	35.3%	0.0%	0.0%	0.0%	32
b.58.Meet other people to do dog activity, e.g. walk together	0.0%	3.2%	0.0%	0.5%	0.0%	0.0%	0.0%	0.0%	0.0%	0.0%	0.0%	3.3%	0.0%	0.0%	0.0%	0.0%	0.0%	5.9%	0.0%	0.0%	0.0%	7
c.59.Talk about the dog, e.g. main subject	0.0%	0.0%	1.8%	2.2%	1.4%	2.9%	0.0%	0.0%	0.0%	0.0%	0.0%	18.9%	0.0%	3.6%	0.0%	0.0%	0.0%	0.0%	0.0%	0.0%	0.0%	34
d.60.Educating people about dogs, e.g., talking, writing book, training	0.0%	0.0%	0.0%	0.0%	0.0%	0.0%	0.0%	0.0%	0.0%	1.1%	2.9%	2.5%	2.3%	1.8%	0.0%	0.0%	0.0%	0.0%	0.0%	0.0%	0.0%	10
e.Interactions on social media, e.g., talking to others, posting pictures																						0
61.Being part of a dog group/talk to other dog owners on social media	0.0%	0.0%	0.0%	0.0%	1.4%	0.0%	0.0%	0.0%	0.0%	0.0%	2.9%	1.6%	0.8%	0.0%	0.0%	0.0%	0.0%	0.0%	0.0%	0.0%	0.0%	6
62.Having a profile for the dog on social media	0.0%	0.0%	0.6%	0.5%	0.0%	0.0%	0.0%	0.0%	0.0%	0.0%	0.0%	0.8%	0.0%	0.0%	0.0%	0.0%	0.0%	0.0%	0.0%	0.0%	0.0%	3
63.Post dog picture/video on social media, read comments on picture/video	0.0%	3.2%	1.8%	1.1%	0.0%	0.0%	50.0%	0.0%	0.0%	0.0%	0.0%	4.1%	0.0%	0.0%	0.0%	0.0%	0.0%	5.9%	0.0%	0.0%	0.0%	13
64.Seeing pictures/videos of dogs on social media, internet	0.6%	0.0%	1.2%	0.5%	0.0%	0.0%	0.0%	0.0%	0.0%	0.0%	0.0%	0.0%	0.0%	0.0%	0.0%	0.0%	0.0%	0.0%	0.0%	0.0%	0.0%	4
VIII.Dog unwanted behaviours/situations																						0
a.Sensory related, e.g., barking, housesoiling, smelly/dirty dog, snoring																						
65.Barking	10.9%	3.2%	0.0%	0.0%	0.0%	0.0%	0.0%	0.0%	0.0%	0.0%	0.0%	0.8%	0.0%	0.0%	0.0%	0.0%	0.0%	0.0%	0.0%	0.0%	0.0%	21
66.Crying, e.g. puppy crying without owner	1.1%	3.2%	0.0%	0.0%	0.0%	0.0%	0.0%	0.0%	0.0%	0.0%	0.0%	0.0%	0.0%	0.0%	0.0%	0.0%	0.0%	0.0%	0.0%	0.0%	0.0%	3
67.Dog or house becomes dirty/smelly, e.g., housesoiling, rolls in fox poo	6.3%	3.2%	0.0%	0.0%	0.0%	0.0%	0.0%	0.0%	0.0%	0.0%	0.0%	0.0%	0.0%	0.0%	0.0%	20.0%	0.0%	0.0%	0.0%	0.0%	0.0%	13
68.Snoring	0.0%	0.0%	0.6%	0.0%	0.0%	0.0%	0.0%	0.0%	0.0%	0.0%	0.0%	0.0%	0.0%	0.0%	0.0%	0.0%	0.0%	0.0%	0.0%	0.0%	0.0%	1
b.Lack of or little control over the dog, e.g., poor response to recall, pulls on the lead																						
69.Jumping up	1.7%	0.0%	0.6%	0.5%	0.0%	2.9%	0.0%	0.0%	0.0%	0.0%	0.0%	0.0%	0.0%	0.0%	0.0%	0.0%	0.0%	0.0%	0.0%	0.0%	0.0%	6
70.Misbehaving - general, e.g., not doing what it is told/expected	3.4%	6.5%	0.0%	0.0%	0.0%	0.0%	0.0%	0.0%	0.0%	0.0%	0.0%	0.0%	0.0%	0.0%	0.0%	20.0%	33.3%	0.0%	0.0%	50.0%	0.0%	12
71.Out of control outdoors, e.g., chasing animal, running away, escaping	2.9%	0.0%	0.0%	0.0%	0.0%	0.0%	0.0%	0.0%	0.0%	0.0%	0.0%	0.0%	0.0%	0.0%	25.0%	0.0%	0.0%	0.0%	0.0%	0.0%	0.0%	7
72.Pulling on the lead	4.6%	3.2%	0.0%	0.0%	0.0%	0.0%	0.0%	0.0%	0.0%	0.0%	0.0%	0.0%	0.0%	0.0%	0.0%	20.0%	0.0%	0.0%	0.0%	25.0%	0.0%	11
73.Unpredictable behaviour	1.7%	3.2%	0.0%	0.0%	0.0%	0.0%	0.0%	0.0%	0.0%	0.0%	0.0%	0.8%	0.0%	0.0%	0.0%	0.0%	0.0%	0.0%	0.0%	0.0%	0.0%	5
74.Not responding to the recall	6.3%	3.2%	0.0%	0.0%	0.0%	0.0%	0.0%	0.0%	0.0%	0.0%	0.0%	0.0%	0.0%	0.0%	0.0%	0.0%	33.3%	0.0%	0.0%	25.0%	0.0%	14
c.Disruption of owner's personal space/moment, e.g., dog seeking attention																						
75.Dog breaks into rooms, e.g., opening doors with paws	0.6%	0.0%	0.6%	0.0%	0.0%	0.0%	0.0%	0.0%	0.0%	0.0%	0.0%	0.0%	0.0%	0.0%	0.0%	0.0%	0.0%	0.0%	0.0%	0.0%	0.0%	2
76.Dog demanding things, e.g., treats, walk, play, attention, objects	4.0%	0.0%	0.0%	0.0%	0.0%	0.0%	0.0%	0.0%	0.0%	0.0%	0.0%	0.0%	0.0%	0.0%	0.0%	0.0%	0.0%	0.0%	0.0%	0.0%	0.0%	7
d. Unruly behaviours, e.g., destruction of objects, hyperactivity																						
77.Dog destroying, throwing, stealing objects or food items	2.3%	0.0%	1.2%	0.0%	0.0%	0.0%	0.0%	33.3%	0.0%	0.0%	0.0%	0.0%	0.0%	0.0%	0.0%	20.0%	0.0%	0.0%	0.0%	0.0%	0.0%	8
78.PICA, e.g., dog eating screws	0.6%	0.0%	0.0%	0.0%	0.0%	0.0%	0.0%	0.0%	0.0%	0.0%	0.0%	0.0%	0.0%	0.0%	0.0%	0.0%	0.0%	0.0%	0.0%	0.0%	0.0%	1
79.Puppy unruly behaviour, e.g.,biting for play	1.7%	0.0%	0.6%	0.0%	0.0%	0.0%	0.0%	33.3%	0.0%	0.0%	1.5%	0.0%	0.0%	0.0%	0.0%	0.0%	0.0%	0.0%	0.0%	0.0%	0.0%	6
80.Hyperactive behaviour, e.g., running around the house, puppy energy	1.1%	0.0%	0.0%	0.0%	0.0%	0.0%	0.0%	0.0%	0.0%	0.0%	0.0%	0.0%	0.0%	0.0%	0.0%	0.0%	0.0%	0.0%	0.0%	0.0%	0.0%	2
e. Fearful/aggressive behaviours, e.g., afraid of noise, biting other dogs																						
81.Dog afraid of something, e.g.,noise, brush	1.7%	3.2%	0.0%	0.0%	0.0%	0.0%	0.0%	0.0%	0.0%	0.0%	0.0%	0.0%	0.0%	0.0%	0.0%	0.0%	0.0%	0.0%	0.0%	0.0%	0.0%	4
82.Dog biting, growling, lunging, fighting with other individuals	2.3%	3.2%	0.0%	0.0%	0.0%	0.0%	0.0%	0.0%	0.0%	0.0%	0.0%	0.0%	0.0%	0.0%	0.0%	0.0%	0.0%	11.8%	0.0%	0.0%	0.0%	7
f.Other: poor appetite, separation related problems, sniffing too much in walks																						
83.Dog not willing to eat, e.g., does not like the food	0.6%	0.0%	0.0%	0.0%	0.0%	0.0%	0.0%	0.0%	0.0%	0.0%	0.0%	0.0%	0.0%	0.0%	0.0%	0.0%	0.0%	0.0%	0.0%	0.0%	0.0%	1
84.Dog separation related problems	0.6%	0.0%	0.0%	0.0%	0.0%	0.0%	0.0%	0.0%	0.0%	0.0%	0.0%	0.0%	0.0%	0.0%	0.0%	0.0%	0.0%	0.0%	0.0%	0.0%	0.0%	1
85.Dog sniffing too much during walk	0.6%	0.0%	0.0%	0.0%	0.0%	0.0%	0.0%	0.0%	0.0%	0.0%	0.0%	0.0%	0.0%	0.0%	0.0%	0.0%	0.0%	0.0%	0.0%	0.0%	0.0%	1
**Number of mentions**	**175**	**31**	**167**	**182**	**70**	**34**	**2**	**3**	**47**	**95**	**68**	**122**	**133**	**55**	**8**	**5**	**3**	**17**	**1**	**4**	**21**	**1243**

0.0%	2.5%	5.0%	7.5%	10.0%	12.5%	15.0%	17.5%	20.0%	22.5%	25.0%	27.5%	30.0%

Figure S1. Heat map and number of mentions of 85 dog-human related activities and their reported well-being outcomes. Darker cells represent a higher proportion of mentions of an activity (row) in a respective well-being outcome (column). E.g., 'the presence of the dog' (dark cell) was mentioned to increase autonomy more times than 'training the dog' (light cell). N and P (negative and positive valence, respectively), H and L (high and low arousal, respectively), Au (autonomy), En (environmental mastery), Pe (personal growth), Pu (purpose in life), Po (positive relations), Se (self-acceptance), LS (life satisfaction increase).

Interview guide/script

<u>Welcome (5 min)</u>

- Hello _______. Thank you for joining this interview!
- My name is Ana Maria Barcelos, I am a PhD student at the University of Lincoln.
- As you saw on the information sheet, we are investigating how dog ownership impacts on the well-being of autistic dog owners.
- Today we will talk about the activities you and your dog do and how they influence your mental health.
- The meeting will be audio recorded, but all the information you share here will be anonymised so you don't need to worry about being honest with us.
- Your Amazon voucher will be sent to you via email after this session.
- Feel free to use the toilet during our conversation or ask for a break at any point. Our interview should take around 30 minutes.
- You are unique and your opinion is very important to the research.
- There is no right or wrong, feel free to share your experiences as a dog owner.
- The consent form is already signed. Do you have any question before we start?
- Start audio recording.

<u>Explanation about what you mean by direct and indirect dog-related activities - PPT slides (2 min)</u>
Any questions?

<u>Explanation about the four aspects of hedonic well-being and life satisfaction - PPT slides (3 min)</u>
Any questions?

<u>Exercise 1 (10 min):</u>
Now, I would like you to think about **the four most important dog-related activities to these aspects of well-being (your feelings and your life satisfaction).** You can write the activities and their well-being outcomes on a piece of paper to help you organise your ideas

*Remind them that they can talk about both direct and indirect activities, and about positive and negative activities, there are no limitations.
*Ask additional questions to clarify the activity/well-being outcome described by the participant

<u>Explanation about the six elements of eudaimonic well-being - PPT slides (3min)</u>
Any questions?

<u>Exercise 2 (10 min):</u>
Now, we will do something similar to the first exercise, but now I would like you to think about these new elements of well-being. Can you think about **the four most important dog-related activities associated with one or more elements**? You can write the activities and well-being outcomes on a piece of paper if that helps.

*Remind them that they can talk about both direct and indirect activities, and about positive and negative activities, there are no limitations.
*Ask additional questions to clarify the activity/well-being outcome described by the participant

Thank you and further information (2 min)

- It was a pleasure to meet you.
- Thank you again for joining this study.
- We had a very nice discussion about dog-related activities and well-being.
- I am very happy with the information you provided, I am sure it will be very useful to our research.
- I will send you the Amazon voucher via email in a few minutes.
- Do you have any question before we finish the call?
- Bye. Have a lovely day.
